# Efficacy and safety of pain management in perioperative period of TACE in patients with HCC: a single-center, parallel-group, randomized trial

**DOI:** 10.3389/fmed.2026.1862804

**Published:** 2026-08-04

**Authors:** Weiren Liang, Zheng Yao, Chaoyi Qian, Jiaping Zheng, Yamin Ma, Jie Chai, Zhehan Bao, Shuwang Chen, Jun Luo

**Affiliations:** Department of Interventional Therapy, Zhejiang Cancer Hospital, Hangzhou Institute of Medicine (HIM), Chinese Academy of Sciences, Hangzhou, Zhejiang, China

**Keywords:** hepatocellular carcinoma, pain, patient-controlled analgesia, quality of life, transcatheter arterial chemoembolization

## Abstract

**Objective:**

To evaluate the efficacy and safety of proactive perioperative pain management with hydromorphone patient-controlled analgesia (PCA) versus standard-care subcutaneous morphine (SCM) in patients with hepatocellular carcinoma undergoing transarterial chemoembolization (TACE).

**Methods:**

This single-center, parallel-group randomized trial enrolled 110 patients, who were assigned 1:1 to hydromorphone PCA or as-needed SCM. The modified intention-to-treat population included 105 patients (PCA, n = 51; SCM, n = 54). Numerical rating scale (NRS) pain scores were assessed intraoperatively and at 2, 12, 24, and 48 h after TACE. The prespecified primary outcome was the 24-h NRS score. Secondary outcomes included clinically relevant pain events, 48-h pain area under the curve (AUC), rescue opioid use, adverse events, quality of life, and factors associated with breakthrough pain.

**Results:**

At 24 h, the mean NRS score was lower in the PCA group than in the SCM group (0.39 ± 0.67 vs. 1.04 ± 1.43; mean difference, −0.64; 95% CI, −1.07, −0.22; Hedges’ g = −0.570; *p* = 0.004). NRS scores ≥4 at any assessment occurred in 21.6% versus 50.0% of patients (risk difference, −28.4 percentage points; 95% CI, −45.9, −11.0; Holm-adjusted *p* = 0.005). The PCA group also had a lower 48-h pain AUC (20.71 ± 28.11 vs. 46.54 ± 52.33 NRS·h; mean difference, −25.83; 95% CI, −41.93, −9.73; Holm-adjusted *p* = 0.005) and less rescue opioid use (3.9% vs. 50.0%; risk difference, −46.1 percentage points; 95% CI, −60.4, −31.7; Holm-adjusted *p* < 0.001). Dizziness was more frequent with PCA (31.4% vs. 3.7%; p < 0.001), whereas overall adverse-event incidence was similar. Quality of life and postoperative hospital stay did not differ significantly. Complete embolization and SCM were associated with breakthrough pain.

**Conclusion:**

Hydromorphone PCA reduced short-term pain burden and rescue opioid requirements after TACE but increased dizziness. Because absolute differences in mean NRS scores were modest, these findings should be interpreted alongside clinically oriented secondary outcomes and the need for individualized safety monitoring.

## Introduction

Transcatheter Arterial Chemoembolization (TACE) is a standard local therapeutic modality for advanced hepatocellular carcinoma (HCC), which can significantly prolong the survival time of patients (1–3). However, despite the crucial role of TACE in treatment, its postoperative complications cannot be ignored, especially postoperative pain. Studies have reported that the incidence of postoperative pain symptoms such as abdominal pain in patients undergoing TACE is as high as 55.6% or more; while the incidence of post-embolization syndrome (PES)—a complex symptom accompanied by fever, nausea and vomiting—even exceeds 20% (4–6). These adverse reactions not only lead to a significant decline in patients’ quality of life, increase the length of hospital stay and medical costs, but also may reduce patients’ compliance with TACE treatment due to severe pain, and even result in treatment interruption caused by intolerable pain (4, 6, 7). At present, although clinical practice routinely uses drugs such as dexamethasone and lidocaine for symptomatic treatment (4, 8), there is still a lack of unified standards for the optimal analgesic management strategy for postoperative pain, especially in balancing effective pain relief and reducing drug side effects (e.g., respiratory depression, constipation, nausea and vomiting), there remains a significant clinical gap.

Management strategies for post-TACE pain are mainly divided into two categories: preventive drug therapy and symptomatic treatment. Preventive drug therapy primarily uses steroidal anti-inflammatory drugs or non-steroidal anti-inflammatory drugs to alleviate pain caused by inflammatory responses (9); while symptomatic treatment mainly relies on opioid drugs (10). Traditional standard care morphine (SCM) analgesia, as a classic supportive treatment method, is effective in pain control, but it is characterized by high administration frequency, slow onset of action and strong dependence, often leading to adverse reactions such as respiratory depression, nausea and vomiting in patients, and requires repeated operations by nurses, imposing a heavy burden on nursing work (9, 11). In recent years, patient-controlled analgesia (PCA) technology has attracted attention because it allows patients to self-administer analgesics immediately when pain occurs. Studies have confirmed that hydromorphone PCA has the advantages of rapid onset, strong analgesic effect and low risk of respiratory depression in controlling cancer pain, and has shown higher patient satisfaction than traditional as-needed administration in postoperative pain management (12). In addition, as a potent opioid analgesic, hydromorphone has high bioavailability and a short half-life, making it suitable for precise dose control via PCA pumps, thereby reducing the risk of side effects caused by drug accumulation. Although hydromorphone PCA has potential advantages in perioperative analgesia for TACE, especially in alleviating the symptoms of post-embolization syndrome and improving quality of life (12, 13), there is currently a lack of specific studies comparing as-needed morphine (SCM) analgesia and hydromorphone PCA in TACE patients.

Therefore, this study intends to compare the postoperative pain control effect, the incidence of post-embolization syndrome and the improvement of quality of life between patients in the hydromorphone PCA group and the as-needed morphine analgesia (SCM) group. Through this study, we aim to clarify whether hydromorphone PCA can more effectively relieve post-TACE pain and reduce the incidence of postoperative adverse reactions, and provide clinicians with a more precise analgesic regimen, thereby improving the overall prognosis and quality of life of patients and filling the current research gap in the field of TACE pain management.

## Materials and methods

### Study subjects

This prospective study was designed as a randomized controlled, parallel-group trial, with a total of 110 patients with HCC enrolled. Patients were randomly assigned at a 1:1 ratio to either the hydromorphone PCA group or the SCM group. All participants were diagnosed with primary hepatocellular carcinoma via clinical or pathological examination, and classified as Barcelona Clinic Liver Cancer (BCLC) stage A-C. This study has been registered with the Chinese Clinical Trial Registry (ChiCTR, www.ChiCTR.org.cn), with the registration number ChiCTR2500110652.

### Inclusion criteria

- Aged 18–80 years.
- Diagnosis of hepatocellular carcinoma confirmed by imaging (contrast-enhanced CT/MRI) or pathology, and scheduled to undergo elective TACE [conventional TACE (cTACE) or drug-eluting bead TACE (dTACE)] in our hospital.
- Barcelona Clinic Liver Cancer (BCLC) stage A–C.
- Eastern Cooperative Oncology Group (ECOG) performance status score of 0–1.
- Child–Pugh class A–B.
- No persistent background pain [resting Numerical Rating Scale (NRS) score ≤ 3] within 7 days before surgery, and no regular use of analgesics [including opioids, non-steroidal anti-inflammatory drugs (NSAIDs), and COX-2 inhibitors].
- Ability to understand and correctly operate the PCA device, and provision of written informed consent.

### Exclusion criteria

- Presence of extrahepatic distant metastases requiring urgent/other anti-tumor treatments.
- A history of past or current regular use of opioid analgesics (≥3 days/week for ≥2 consecutive weeks) or a medical history of moderate to severe chronic pain.
- Known hypersensitivity to hydromorphone, morphine, or contrast agents.
- Severe cardiopulmonary diseases (e.g., NYHA class III–IV heart failure, recent myocardial infarction, severe chronic obstructive pulmonary disease [COPD]/respiratory failure) or a high risk of significant respiratory depression.
- Renal insufficiency: serum creatinine > 176.8 μmol/L or creatinine clearance < 30 mL/min.
- Hematological abnormalities: white blood cell count < 3 × 10^9^/L, hemoglobin < 85 g/L, or platelet count < 50 × 10^9^/L.
- Severe hepatic decompensation (e.g., refractory ascites, active gastrointestinal bleeding, hepatic encephalopathy ≥ grade II) or uncontrolled infection.
- Pregnancy or lactation.
- Other conditions deemed unsuitable for enrollment by the investigators.

### Sample size calculation

The primary outcome of this study was defined as the NRS score at 24 h after TACE. Based on data from pilot experiments and previous similar studies, the expected mean difference in NRS scores at 24 h post-TACE between the two groups was 0.64 points, with a pooled standard deviation of approximately 1.11 points. A two-tailed test was adopted with a significance level (*α*) of 0.05 and a power of test (1-*β*) of 0.80. Calculations for comparing the means of two independent samples indicated that a minimum of 48 participants were required for each group. Considering a potential 10–15% rate of loss to follow-up or withdrawal, this study planned to enroll approximately 55 participants per group, resulting in a total sample size of 110 cases.

### Study randomization

The random allocation sequence was generated by an independent statistician using the random number generator in Microsoft Excel. Allocation concealment was ensured using sequentially numbered, opaque, sealed envelopes prepared by one of the researchers who is not involved in the following treatment procedures for the patients. The allocation sequence was concealed until patients were enrolled and assigned to interventions. Patients, clinicians, and outcome assessors were not blinded. To minimize the impact of interventional operators on the study outcomes, TACE treatment for all enrolled patients was conducted by two designated and well-trained physicians (Dr. LJ and Dr. YZ), both of whom are interventional radiologists and key participants in this study.

Patients with HCC scheduled to undergo TACE at our hospital will be screened and enrolled in the study. All enrolled patients will first complete preoperative examinations, including a complete blood count, coagulation screening, electrocardiogram (ECG), contrast-enhanced abdominal magnetic resonance imaging (MRI), and chest computed tomography (CT). The diagnosis of HCC will be confirmed based on pathological findings or the BCLC clinical diagnostic criteria for hepatocellular carcinoma (1). All patients will be followed up until the PCA device is removed at the end of the treatment period or the occurrence of complications.

## Treatment methods

### TACE procedure

After thorough disinfection and draping, local anesthesia was administered at the puncture site with 2% lidocaine. The femoral artery was accessed via the Seldinger technique, followed by insertion of a 5-French vascular sheath. A 4-French RH catheter was then advanced into the abdominal aorta or superior mesenteric artery for angiographic assessment to identify the tumor-feeding arteries. A 2.6–2.8 Fr microcatheter was selectively guided into the identified tumor-feeding arteries, and embolic agents were gradually infused to achieve embolization. The endpoint of embolization was defined as stasis of antegrade blood flow within the tumor-feeding arteries.

#### Pain management protocol

The acute phase of TACE was defined as 48 h, which was divided into five time intervals: Time 0 (T0) was intraoperative TACE; Time 2 (T2), Time 12 (T12), Time 24 (T24) and Time 48 (T48) were 2 h, 12 h, 24 h and 48 h after T0, respectively. All medications were administered in accordance with the recommended dosages in the manufacturers’ instructions, as detailed below:

Hydromorphone PCA Group: (1) PCA pump preparation and administration 30 min before surgery: 10 mg of hydromorphone hydrochloride injection was mixed with 90 mL of normal saline to prepare the patient-controlled analgesia pump. The pump parameters were set as follows: continuous infusion rate: 0.15–0.3 mg/h; bolus dose: 0.2–0.4 mg; lockout interval: 10 min. (2) Intraoperative intervention: Patients were instructed to press the PCA pump once for a bolus dose before the initiation of embolization. If the NRS score remained ≥ 4 after two consecutive valid presses, a rescue dose of 5 mg morphine was administered subcutaneously. (3) Postoperative maintenance: Continuous infusion combined with patient-controlled bolus administration was maintained for 48 h. If the NRS score remained ≥ 4 after two consecutive valid presses, a rescue dose of 5 mg morphine was administered subcutaneously. SCM Group: (1) No sedative or analgesic medications were administered before surgery. (2) During surgery and postoperatively, 5–10 mg morphine was administered subcutaneously when the NRS score reached ≥ 4.

The use of additional non-steroidal anti-inflammatory drugs (NSAIDs), including COX-2 inhibitors, was prohibited in both groups. Physical cooling therapy was recommended for patients with fever during the study period. Prophylactic medications such as corticosteroids, antiemetics, or anesthetics were not permitted for the management of common TACE-related adverse events (AEs) in this study; however, rescue therapy for post-TACE symptoms was prescribed reasonably by attending physicians.

### Outcome measures

The NRS was adopted in this study to quantitatively assess pain intensity. This scale ranges from 0 (no pain) to 10 (worst imaginable pain) and has good reliability and validity. Pain scores were recorded at five predefined time points: intraoperatively immediately after administration of the chemoembolization agents (T0), and at 2 h (T2), 12 h (T12), 24 h (T24), and 48 h (T48) after TACE. The NRS score at 24 h after TACE was the prespecified primary pain outcome, whereas the intraoperative, 2-h, 12-h, and 48-h assessments were treated as exploratory time-point outcomes. The maximum self-reported NRS score recorded within each predefined assessment window was used for analysis.

Breakthrough pain events were also recorded in this study and were operationally defined as acute pain exacerbations with an NRS score ≥ 4 during the 48-h observation period; the frequency of such events was documented. Rescue analgesic interventions were standardized as follows: when pain control was inadequate (persistent NRS score ≥ 4), patients in the hydromorphone PCA group received a subcutaneous injection of 5 mg morphine as rescue analgesia after two consecutive valid patient-controlled doses, while those in the SCM group received the same rescue regimen after as-needed administration. The frequency and total cumulative dose of rescue morphine administered were recorded for both groups. Several clinically interpretable secondary analgesic endpoints were derived from the five predefined NRS assessments. These included the proportion of patients with at least one NRS score ≥4, the number of moderate-to-severe pain episodes defined as NRS ≥ 4 across the five assessment time points, and the proportion of patients requiring at least one rescue opioid administration. These scheduled-assessment endpoints were analyzed separately from breakthrough pain events, which represented unscheduled acute pain exacerbations recorded between the predefined assessment time points. Overall pain burden during the 48-h observation period was summarized as the area under the NRS–time curve (pain AUC), calculated using the trapezoidal rule. Time to first rescue analgesia was considered clinically relevant but could not be analyzed because the exact timing of rescue administration was not recorded with sufficient completeness and precision.

All patients were monitored for adverse reactions within 7 days postoperatively, with common symptoms including nausea, vomiting, dizziness, fatigue, constipation, anorexia, epigastric discomfort, abdominal distension, and fever documented. The severity of adverse events was graded according to the criteria of the CTCAE Version 5.0, and serious adverse events leading to study discontinuation were also recorded.

In addition, the quality of life of patients was assessed using the Chinese version of the European Organization for Research and Treatment of Cancer Quality of Life Questionnaire Core 30 (EORTC QLQ-C30) on the 7th day postoperatively. EORTC QLQ-C30 is a 30-item instrument including five functional scales, eight symptom scales, a finance item, and a global health QoL scale. Response options were on a 4-point Likert-type scale from ‘not at all’ to ‘very much’ for the functional scales, symptom scales, and the finance item, and a 7-point Likert scale for global health QoL ranging from ‘Very poor’ to ‘Excellent’. In the present study, the global health status/quality-of-life component of the EORTC QLQ-C30 was evaluated using items 29 and 30. Each item was rated on a 7-point scale ranging from 1 (‘very poor’) to 7 (‘excellent’). The mean of the two item scores was calculated and retained on the original 1–7 scale without linear transformation to a 0–100 scale, with a higher score indicating better perceived global health status and quality of life. The postoperative length of hospital stay (defined as the number of days from the end of surgery to discharge) was documented. As a pragmatic proxy for treatment satisfaction, patients’ willingness to undergo TACE again if clinically indicated was assessed using the question: “Would you be willing to receive TACE treatment again if your condition requires it?” Because no validated patient-satisfaction instrument was administered, this variable was interpreted only as a satisfaction proxy rather than as a formal measure of patient satisfaction. All data were collected by trained research assistants using standardized electronic Case Report Forms (eCRFs) and verified by two independent personnel to ensure accuracy.

### Statistical methods

Statistical analyses were performed using SPSS Statistics version 26.0 and Python version 3.9. Continuous variables are presented as mean ± standard deviation (SD), and categorical variables are presented as frequencies and percentages. Between-group comparisons of continuous outcomes measured at a single time point were performed using the independent-samples t-test when appropriate. Categorical variables were compared using the Pearson chi-square test or Fisher’s exact test, as appropriate. Longitudinal NRS pain scores were analyzed using a linear mixed-effects model to account for within-patient correlation across repeated measurements. The model included treatment group, time as a categorical variable, and the treatment group × time interaction as fixed effects, together with a subject-specific random intercept. Model parameters were estimated using restricted maximum likelihood. Overall time, treatment-group, and group × time effects were evaluated using Type III Wald F tests. The intraoperative assessment was used as the reference time category for model parameterization. The NRS score at 24 h after TACE was the prespecified primary pain outcome, and the primary inference was based on its unadjusted *p* value. As a supplementary multiplicity-controlled analysis, *p* values for the four post-TACE assessments at 2, 12, 24, and 48 h were adjusted using the Holm–Bonferroni step-down procedure. The intraoperative comparison was treated as a separate exploratory analysis and was not included in this multiplicity family. Absolute mean differences and Hedges’ g with 95% confidence intervals were reported to quantify treatment effects. For binary endpoints, treatment effects were expressed as absolute risk differences with 95% confidence intervals. For count outcomes, incidence-rate ratios with 95% confidence intervals were estimated using negative binomial regression, with the hydromorphone PCA group as the reference category. Hedges’ g was interpreted descriptively as approximately 0.2, 0.5, and 0.8 for small, moderate, and large standardized effects, respectively. The 48-h pain AUC was calculated by trapezoidal integration of the five recorded NRS values over time. The four prespecified clinically oriented secondary endpoints included the proportion with NRS ≥ 4 at any time point, the 48-h pain AUC, the proportion requiring rescue opioids, and the number of moderate-to-severe pain episodes. Their *p* values were adjusted using the Holm–Bonferroni step-down procedure. Time to first rescue analgesia and the satisfaction proxy were not included in this multiplicity family because the former was unavailable and the latter was considered an ancillary exploratory outcome. Univariate analyses were performed to examine factors associated with breakthrough pain. Variables selected for multivariable logistic regression were entered into the model to identify factors significantly associated with breakthrough pain. Collinearity was evaluated before multivariable analysis. All tests were two-sided, and *p* < 0.05 was considered statistically significant.

## Results

**Figure 1:**
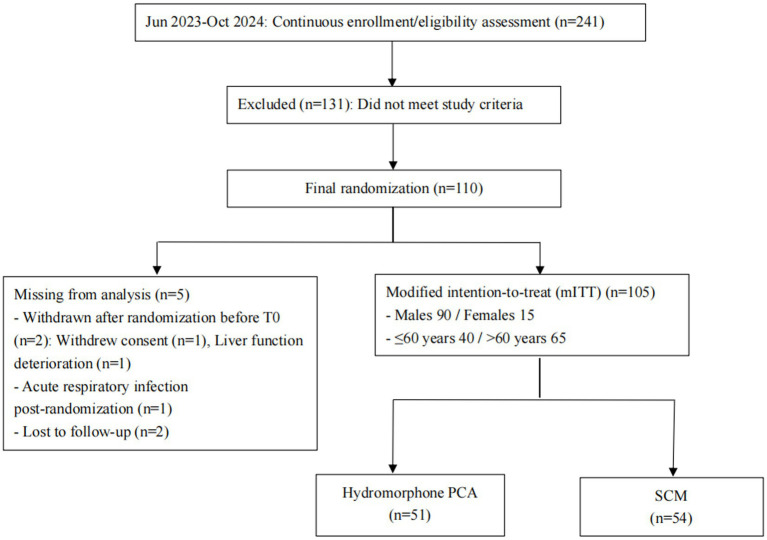
Study flow diagram of patient enrollment and allocation.

Between June 2023 and October 2024, a total of 241 consecutive patients were assessed for eligibility. Of these, 131 did not meet the eligibility criteria. Among the 110 patients who were finally enrolled and randomly assigned, two withdrew from the study before T0 (one withdrew consent and one developed liver function deterioration), one had acute respiratory tract infection after randomization, and two lost to follow-up. A total of 105 patients were included in the modified intention-to-treat (mITT) population. Of these patients, 90 were male and 15 were female; 40 were ≤ 60 years old and 65 were > 60 years old. After randomization, there were 51 patients in the hydromorphone PCA group and 54 patients in the SCM group. The study process is illustrated in Figure 1. There were no statistically significant differences in clinical characteristics between the two groups (*p* > 0.05) (Table 1).

**Table 1:** Comparison of clinical data between the two groups.

Clinical data	Hydromorphone PCA (*n* = 51)	SCM (*n* = 54)	*p* value
Gender			0.873
Male	44 (86.3)	46 (85.2)	
Female	7 (13.7)	8 (14.8)	
Age			0.329
≤60	17 (33.3)	23 (42.6)	
>60	34 (66.7)	31 (57.4)	
BMI			0.690
<24	36 (70.6)	40 (74.1)	
≥24	15 (29.4)	14 (25.9)	
BCLC stage			0.464
A	7 (13.7)	7 (13.0)	
B	29 (56.9)	25 (46.3)	
C	15 (29.4)	22 (40.7)	
Operation			0.683
cTACE	34 (66.7)	38 (70.4)	
dTACE	17 (33.3)	16 (29.6)	
Embolization degree			0.623
Incomplete	24 (47.1)	28 (51.9)	
Complete	27 (52.9)	26 (48.1)	
Distance to liver capsule			0.481
≤1 cm	29 (56.9)	27 (50.0)	
>1 cm	22 (43.1)	27 (50.0)	
Lesion’s number			0.577
<3	19 (37.3)	23 (42.6)	
≥3	32 (62.7)	31 (57.4)	
Lesion’s size			0.675
<10 cm	44 (86.3)	45 (83.3)	
≥10 cm	7 (13.7)	9 (16.7)	
Up to seven			0.566
No	33 (64.7)	32 (59.3)	
Yes	18 (35.3)	22 (40.7)	

Data are presented as *n* (%). BMI, body mass index; BCLC, Barcelona Clinic Liver Cancer; cTACE, conventional transarterial chemoembolization; dTACE, drug-eluting bead transarterial chemoembolization; PCA, patient-controlled analgesia; SCM, standard-care morphine; TACE, transarterial chemoembolization.

Longitudinal NRS pain scores were analyzed using a linear mixed-effects model. A significant overall time effect was observed (*F* = 95.76, *p* < 0.001), indicating that pain scores changed over the five assessment time points. The overall treatment-group effect was also significant (*F* = 10.42, *p* = 0.002), with lower NRS scores across the observation period in the hydromorphone PCA group. However, the treatment group × time interaction was not statistically significant (*F* = 1.38, *p* = 0.240), indicating that the temporal pattern of change in NRS scores did not differ significantly between the two groups (Supplementary Table S1). At the prespecified primary time point of 24 h after TACE, the mean NRS score was lower in the hydromorphone PCA group than in the SCM group (0.39 ± 0.67 vs. 1.04 ± 1.43), with an absolute mean difference of −0.64 points (95% CI, −1.07, −0.22; Hedges’ g = −0.570; *p* = 0.004). The absolute mean difference at 24 h was smaller than the commonly used 1-point reference threshold for a clinically important change in post-procedural NRS pain. Similarly, although the intraoperative and 48-h standardized effects were of moderate magnitude, their absolute mean differences were −0.94 and −0.46 NRS points, respectively (Table 2). In the separate exploratory intraoperative analysis, the hydromorphone PCA group had a lower NRS score than the SCM group. Among the post-TACE assessments included in the supplementary Holm–Bonferroni analysis, the between-group difference remained statistically significant at 48 h, whereas the differences at 2 and 12 h were not statistically significant. The complete time-point-specific results are presented in Table 2. The hydromorphone PCA group also had fewer breakthrough pain episodes, fewer rescue morphine administrations, and a lower cumulative rescue morphine dose than the SCM group (Table 2).

**Table 2:** Comparison of pain-related outcomes between the hydromorphone patient-controlled analgesia group and the subcutaneous morphine group.

Time/endpoint	Hydromorphone PCA (*n* = 51)	SCM (*n* = 54)	Hedges’ g (95% CI)	Δ (95% CI)	Raw p	Holm-adj p
NRS pain score						
In TACE	2.16 ± 1.43	3.09 ± 1.71	−0.588 (−0.992, −0.221)	−0.94 (−1.54, −0.33)	0.003	—
2 h after TACE	0.78 ± 1.10	1.19 ± 1.59	−0.289 (−0.654, +0.100)	−0.40 (−0.92, +0.12)	0.135	0.135
12 h after TACE	0.51 ± 1.07	1.00 ± 1.48	−0.376 (−0.743, +0.002)	−0.49 (−0.98, +0.00)	0.053	0.107
24 h after TACE	0.39 ± 0.67	1.04 ± 1.43	−0.570 (−0.872, −0.211)	−0.64 (−1.07, −0.22)	0.004	0.011
48 h after TACE	0.10 ± 0.30	0.56 ± 0.96	−0.628 (−0.902, −0.332)	−0.46 (−0.73, −0.19)	0.002	0.006
Moderate-to-severe (NRS ≥ 4) pain episodes (count of 5)	0.25 ± 0.52	0.78 ± 1.00	−0.643 (−0.974, −0.328)	−0.52 (−0.83, −0.22)	0.002	—
Times of breakthrough pain	0.24 ± 0.681	0.78 ± 1.269	−0.525 (−0.851, −0.204)	−0.542 (−0.929, −0.156)	0.007	—
Times of Rescue use of morphine	0.04 ± 0.196	0.91 ± 1.483	−0.804 (−1.099, −0.627)	−0.868 (−1.267, −0.469)	<0.001	—
Dose of Rescue use of morphine	0.29 ± 1.553	5.74 ± 10.159	−0.734 (−1.114, −0.558)	−5.447 (−8.190, −2.704)	<0.001	—

Continuous variables are presented as mean ± standard deviation (SD). The NRS score at 24 h after TACE was the prespecified primary pain outcome, and its raw p value was used for the primary inference. Holm-adjusted p values are additionally presented for the four post-TACE assessments at 2, 12, 24, and 48 h. The intraoperative comparison was not included in this multiplicity family. Treatment effects were quantified using absolute mean differences and Hedges’ g with 95% confidence intervals (CIs). Negative values indicate lower values in the hydromorphone PCA group compared with the SCM group. Breakthrough pain episodes were defined as episodes with an NRS score ≥4 during the predefined observation period. Rescue morphine refers to additional opioid administration required for inadequate pain control. NRS, numerical rating scale; PCA, patient-controlled analgesia; SCM, subcutaneous morphine; CI, confidence interval; TACE, transarterial chemoembolization.

Clinically oriented secondary analgesic outcomes are summarized in Table 3. At least one NRS score ≥4 was recorded in 11 of 51 patients (21.6%) in the hydromorphone PCA group and 27 of 54 patients (50.0%) in the SCM group, corresponding to an absolute risk difference of −28.4 percentage points (95% CI, −45.9, −11.0; Holm-adjusted *p* = 0.005). The 48-h pain AUC was lower in the hydromorphone PCA group than in the SCM group (20.71 ± 28.11 vs. 46.54 ± 52.33 NRS·h), with a mean difference of −25.83 NRS·h (95% CI, −41.93, −9.73; Hedges’ g = −0.600; Holm-adjusted p = 0.005). Rescue opioids were required by 2 of 51 patients (3.9%) in the hydromorphone PCA group and 27 of 54 patients (50.0%) in the SCM group, yielding an absolute risk difference of −46.1 percentage points (95% CI, −60.4, −31.7; Holm-adjusted *p* < 0.001). The number of moderate-to-severe pain episodes was also lower in the hydromorphone PCA group (0.25 ± 0.52 vs. 0.78 ± 0.99); the estimated incidence rate in the SCM group was 3.051 times that in the PCA group (95% CI, 1.470, 6.334; Holm-adjusted *p* = 0.005). Time to first rescue analgesia could not be evaluated because reliable timing data were unavailable. No statistically significant between-group difference was observed in unwillingness to undergo the same procedure again, which was used only as a proxy for treatment satisfaction (0.0% vs. 3.7%; risk difference, −3.7 percentage points; 95% CI, −8.7, 1.3; *p* = 0.165).

**Table 3:** Clinically oriented secondary analgesic outcomes between the hydromorphone patient-controlled analgesia group and the subcutaneous morphine group.

Endpoint	Hydromorphone PCA (*n* = 51)	SCM (*n* = 54)	Standardised effect size (95% CI)	Absolute effect size (95% CI)	Raw p	Holm-adj p
Proportion with NRS ≥ 4 (any time point)	11/51 (21.6%)	27/54 (50.0%)	—	−0.284 (−0.459, −0.110) risk difference	0.002	0.005
48 h trapezoidal pain AUC (NRS·h)	20.71 ± 28.11	46.54 ± 52.33	−0.600 (−0.938, −0.240) Hedges’ g	−25.83 (−41.93, −9.73) mean difference	0.002	0.005
Proportion requiring rescue opioid	2/51 (3.9%)	27/54 (50.0%)	—	−0.461 (−0.604, −0.317) risk difference	<0.001	<0.001
Time to first rescue analgesia	data not available	data not available	—	—	—	not part of multiplicity family
Unwillingness to undergo the same procedure (satisfaction proxy)	0/51 (0.0%)	2/54 (3.7%)	—	−0.037 (−0.087, +0.013) risk difference	0.165	not part of multiplicity family
Moderate-to-severe (NRS ≥ 4) pain episodes (count of 5)	0.25 ± 0.52	0.78 ± 0.99	−0.643 (−0.990, −0.308) Hedges’ g	3.051 (1.470, 6.334) incidence-rate ratio	0.002	0.005

Binary outcomes are presented as numbers and percentages, and treatment effects are expressed as absolute risk differences with 95% confidence intervals (CIs). The 48-h pain area under the curve (AUC) was calculated using the trapezoidal method based on the predefined NRS measurements. Moderate-to-severe pain episodes were defined as the number of assessment time points with an NRS score ≥4 during the 48-h observation period. Incidence-rate ratios (IRRs) for pain episodes were estimated using negative binomial regression, with the SCM group compared with the hydromorphone PCA group. Unwillingness to undergo the same procedure again was assessed as a pragmatic proxy for treatment satisfaction rather than a validated satisfaction measure. *p* values for clinically oriented secondary endpoints were adjusted using the Holm–Bonferroni step-down procedure where applicable. NRS, numerical rating scale; AUC, area under the curve; IRR, incidence-rate ratio; PCA, patient-controlled analgesia; SCM, subcutaneous morphine; CI, confidence interval; TACE, transarterial chemoembolization.

A total of one serious adverse event (Grade 3) occurred throughout the study, which was observed in the hydromorphone PCA group. The PCA pump was discontinued due to severe nausea and vomiting, and the affected patient was female with a low body mass index (BMI). All other adverse reactions were mild or moderate (Grade 1–2), including nausea and vomiting, dizziness, fatigue, fever, constipation, anorexia, epigastric discomfort, and abdominal distension. The incidence of dizziness in the hydromorphone PCA group was 31.4% (16/51), which was significantly higher than that in the SCM group (3.7%, 2/54) (*p* < 0.05). However, there were no statistically significant differences between the two groups in terms of other adverse reactions and the total number of patients with adverse events (*p* > 0.05) (Table 4).

**Table 4:** Adverse events of two groups.

AE	Hydromorphone PCA (*n* = 51)	SCM (*n* = 54)	*p* value
Nausea and vomiting	13 (25.5)	16 (29.6)	0.635*
Dizziness	16 (31.4)	2 (3.7)	<0.001*
Weak	9 (17.6)	11 (20.4)	0.722*
Fever	7 (13.7)	8 (14.8)	0.873*
Constipation	6 (11.8)	4 (7.4)	0.519**
Loss of appetite	4 (7.8)	4 (7.4)	1.000**
Stomach discomfort	3 (5.9)	4 (7.4)	1.000**
Abdominal distension	3 (5.9)	9 (16.7)	0.083*
Patients with any adverse event	38 (74.5)	40 (74.1)	0.959*

Data are presented as *n* (%). “Patients with any adverse event” refers to patients who experienced at least one adverse event during the 7-day postoperative observation period rather than the cumulative number of adverse-event episodes. * Pearson’s Chi-square test; **Fisher’s Exact Test. AE, adverse event; PCA, patient-controlled analgesia; SCM, standard-care morphine; TACE, transarterial chemoembolization.

There were no statistically significant between-group differences in the categorized QoL score, the mean raw global health/QoL score derived from EORTC QLQ-C30 items 29 and 30 at 7 days postoperatively, postoperative length of hospital stay, or willingness to undergo TACE again (all *p* > 0.05) (Table 5).

**Table 5:** Comparison of QOL scores, postoperative hospital stay, willingness to reoperation and EORTC scores between the two groups.

Variables	Hydromorphone PCA (*n* = 51)	SCM (*n* = 54)	*p* value
QoL scores			0.517*
>40	38 (74.5)	35 (64.8)	
>30 and ≤40	11 (21.6)	15 (27.8)	
≤30	2 (3.9)	4 (7.4)	
Postoperative hospital stay (day)	2.67 ± 0.99	2.87 ± 0.87	0.265
Willingness to be reoperation (yes)	51 (100)	52 (96.3)	0.492**
EORTC QLQ-C30 raw global health/QoL score at 1 week	5.90 ± 0.73	5.91 ± 0.71	0.969

Categorical variables are presented as *n* (%), and continuous variables are presented as mean ± standard deviation. * Pearson’s Chi-square test; **Fisher’s Exact Test Continuous outcomes were compared using the independent-samples t-test. EORTC QLQ-C30, European Organisation for Research and Treatment of Cancer Quality of Life Questionnaire Core 30; QoL, quality of life; PCA, patient-controlled analgesia; SCM, standard-care morphine; TACE, transarterial chemoembolization.

As shown in Table 6, univariate analysis showed that the proportion of complete embolization and SCM analgesia in patients with breakthrough pain was significantly higher than that without breakthrough pain (*p* < 0.05).

**Table 6:** Univariate analysis of breakthrough pain.

Variables	No breakthrough pain (*n* = 73)	Breakthrough pain (*n* = 32)	*p* value
Gender			0.387
Male	64 (87.7)	26 (81.3)	
Female	9 (12.3)	6 (18.7)	
Age			0.934
≤60	28 (38.4)	12 (37.5)	
>60	45 (61.6)	20 (62.5)	
BMI			0.178
<24	50 (68.5)	26 (81.3)	
≥24	23 (31.5)	6 (18.7)	
BCLC stage			0.158
A	9 (12.3)	5 (15.6)	
B	42 (57.5)	12 (37.5)	
C	22 (30.1)	15 (46.9)	
Operation			0.072
cTACE	54 (74.0)	18 (56.3)	
dTACE	19 (26.0)	14 (43.7)	
Embolization degree			0.013
Incomplete	42 (57.5)	10 (31.3)	
Complete	31 (42.5)	22 (68.8)	
Distance to liver capsule			0.692
≤1 cm	38 (52.1)	18 (56.3)	
>1 cm	35 (47.9)	14 (43.7)	
Lesion’s number			0.166
<3	26 (35.6)	16 (50.0)	
≥3	47 (64.4)	16 (50.0)	
Lesion’s size			0.942
<10 cm	62 (84.9)	27 (84.4)	
≥10 cm	11 (15.1)	5 (15.6)	
Up to seven			0.22
No	48 (65.8)	17 (53.1)	
Yes	25 (34.2)	15 (46.9)	
Analgesic method			<0.001
Hydromorphone PCA	44 (60.3)	7 (21.9)	
SCM	29 (39.7)	25 (78.1)	

Data are presented as *n* (%). *p* values were calculated using the Pearson chi-square test or Fisher’s exact test, as appropriate. BMI, body mass index; BCLC, Barcelona Clinic Liver Cancer; cTACE, conventional transarterial chemoembolization; dTACE, drug-eluting bead transarterial chemoembolization; PCA, patient-controlled analgesia; SCM, standard-care morphine; TACE, transarterial chemoembolization.

Based on the results of the univariate analysis, the proportion of complete embolization and SCM analgesia were entered to the multivariate model. As shown in Table 7, the results of multivariate analysis indicated that completed embolization [odds ratio (OR) = 3.945; 95% confidence interval (CI): 1.488–10.458; *p* = 0.006] and SCM administration (OR = 6.711; 95% CI: 2.410, 18.685; *p* < 0.001) were significantly associated with breakthrough pain.

**Table 7:** Multivariate analysis of breakthrough pain.

Variables	*B*	S.E.	*Wald χ^2^*	*p*	OR	95%CI
Embolization_degree-complete	1.373	0.497	7.616	0.006	3.945	1.488, 10.458
Analgesic method-SCM	1.904	0.522	13.279	<0.001	6.711	2.410, 18.685

Multivariable logistic regression was performed with breakthrough pain as the dependent variable. Incomplete embolization and hydromorphone PCA were used as the reference categories for embolization extent and analgesic strategy, respectively. B, regression coefficient; SE, standard error; OR, odds ratio; CI, confidence interval; PCA, patient-controlled analgesia; SCM, standard-care morphine.

## Discussion

This study systematically evaluated the efficacy and safety of proactive pain management using hydromorphone PCA versus traditional as-needed morphine analgesia for perioperative pain management in patients undergoing TACE by adopting a prospective randomized controlled design. The longitudinal analysis showed significant overall time and treatment-group effects on NRS pain scores, but no significant treatment group × time interaction. At the prespecified 24-h time point, the hydromorphone PCA group had a lower mean NRS score than the SCM group. Exploratory analyses also showed lower NRS scores intraoperatively and at 48 h after multiplicity adjustment. In addition, the hydromorphone PCA strategy was associated with fewer breakthrough pain episodes and reduced rescue morphine use. Despite a higher incidence of dizziness in the PCA group, the overall incidence of adverse reactions was comparable between the two groups, indicating an acceptable safety profile of hydromorphone PCA. In addition, completed embolization and SCM analgesia were significantly associated with breakthrough pain.

The findings suggest that the proactive hydromorphone PCA strategy may reduce pain-related treatment burden, particularly by decreasing rescue analgesic requirements and clinically relevant pain events. The observed analgesic differences may partly be explained by the pharmacological properties of hydromorphone and the patient-controlled mode of administration. As a semisynthetic derivative of morphine, hydromorphone has higher liposolubility and stronger analgesic potency (14–16). This is consistent with the findings of existing studies reporting that hydromorphone administered via intravenous PCA can maintain more stable blood drug concentrations and reduce analgesic fluctuations (17). Furthermore, hydromorphone has a rapid onset of action (approximately 15 min after intravenous injection) (14). Combined with the patient-controlled dosing mode of PCA pumps, it can promptly alleviate pain upon perception, thereby effectively reducing the incidence of postoperative breakthrough pain (14). Although the standardized between-group effects at the significant time points were generally of moderate magnitude, the absolute differences in mean NRS scores were modest. At the prespecified 24-h time point, the mean difference was −0.64 NRS points, and the absolute differences at the other statistically significant time points were also less than 1 point. None of these point estimates clearly exceeded the commonly used 1-point reference threshold for a clinically important difference in post-procedural NRS pain. Therefore, the statistically significant differences in mean NRS scores should not, by themselves, be interpreted as establishing a clearly perceptible clinical benefit.

The lower 48-h pain AUC and fewer moderate-to-severe pain episodes further suggested reductions in cumulative pain burden and clinically relevant pain exacerbations, outcomes that may be more directly meaningful to patients and clinicians than small differences in mean NRS scores at isolated time points. However, because these analyses were secondary and some were added to improve the clinical interpretation of the trial findings, they should be regarded as supportive rather than definitive evidence.

Regarding the risk factor analysis of breakthrough pain, logistic regression in this study revealed that completed embolization and SCM analgesia were significantly associated with breakthrough pain, among which SCM analgesia was associated with the most significant increase in risk. The pain caused by completed embolization mainly stems from the ischemic necrosis of tumor tissues and the inflammatory response of surrounding tissues (18). In contrast, SCM is characterized by large fluctuations in drug concentrations, which makes it difficult to timely cover the rapidly escalating pain perception, thus easily leading to the occurrence of breakthrough pain (14, 19). This result also verifies the viewpoint reported in existing literature that “as-needed analgesia is difficult to maintain stable blood drug concentrations and is prone to sudden pain exacerbation” (18, 19). Therefore, for procedures accompanied by severe ischemic pain such as TACE, the application of proactive pain management using hydromorphone PCA can significantly reduce the risk of breakthrough pain.

In terms of safety, there was no significant difference in the overall incidence of adverse reactions between the two groups, suggesting that proactive pain management using hydromorphone PCA is well-tolerated in TACE patients. However, the incidence of dizziness in the hydromorphone PCA group was significantly higher than that in the SCM group, which reminds clinicians to pay attention to individualized dosing and adverse reaction monitoring in clinical practice. Although hydromorphone has a low risk of respiratory depression, its high liposolubility may still cause central nervous system side effects in some patients (20, 21). Only one grade 3 adverse event, consisting of severe nausea and vomiting requiring PCA discontinuation, was documented. No respiratory depression was documented; however, incomplete sedation and respiratory monitoring data precluded a definitive assessment of respiratory safety. This is consistent with the conclusion of existing literature that hydromorphone PCA has a favorable safety profile in cancer patients (22–24). Therefore, clinicians can further optimize the tolerability of hydromorphone PCA by adjusting the basal infusion rate or combining it with antiemetic drugs (e.g., dexamethasone) (18, 25).

Nevertheless, no statistically significant between-group differences were observed in the raw global health/QoL score derived from EORTC QLQ-C30 items 29 and 30 at 7 days postoperatively, postoperative length of hospital stay, or willingness to undergo TACE again. Although hydromorphone PCA was associated with lower pain scores, these differences did not translate into significant improvements in the raw global health/QoL score or postoperative length of hospital stay. This may be attributed to the following factors. First, the postoperative length of hospital stay after TACE has been relatively short in most hospitals in China, and it is affected by various factors (e.g., underlying diseases, complications). Thus, pain control alone is difficult to significantly shorten the length of hospital stay (26–28). Second, perceived global health and quality of life are influenced by multiple dimensions, including pain, emotional status, and physical functioning (29, 30). Although the PCA group had lower pain scores, differences in emotional status and functional recovery may have been limited, which could explain the absence of a between-group difference in the raw global health/QoL score (31, 32). This suggests that future studies should consider designing multimodal analgesic regimens (e.g., combining with non-steroidal anti-inflammatory drugs or nerve block) to improve both pain control and overall QoL of patients.

The strengths of this study lie in that it is the first to systematically evaluate the analgesic mode of hydromorphone PCA during the TACE perioperative period, and it identified SCM analgesia and completed embolization were significantly associated with breakthrough pain through multivariate regression analysis, providing an evidence-based basis for clinical analgesic regimen selection. However, this study also has certain limitations. First, the sample size was limited and patients were limited to ECOG 0–1 and Child-Pugh A-B, so it was unable to explore the differences in analgesic efficacy among different doses or basal infusion rates, and the findings may not apply to frailer HCC patients, other hospitals, or different TACE protocols. Second, data on variables such as baseline anxiety and baseline QoL were not collected; the absence of baseline data on anxiety and QoL weakens the postoperative comparison between groups. Consequently, these could not be adjusted for in the regression model, which may lead to residual confounding and affect the model’s accuracy. Third, the exact time of each rescue analgesic administration was not recorded with sufficient completeness and precision; therefore, time to first rescue analgesia could not be evaluated. In addition, patient satisfaction was not assessed using a validated instrument. Willingness to undergo the same procedure again was used only as a pragmatic proxy and may not capture the multidimensional construct of treatment satisfaction. Several clinically oriented secondary endpoints were added in the revised analysis to improve interpretation of clinical relevance; these findings should therefore be considered supportive and exploratory. Fourth, the safety assessment was limited by incomplete documentation of standardized sedation scores and the absence of systematically reported respiratory monitoring variables, such as respiratory rate and oxygen saturation. Although no respiratory depression was documented, the available data were insufficient to establish the respiratory safety of hydromorphone PCA. Fifth, no long-term follow-up was conducted, so the impact of hydromorphone PCA on long-term QoL and chronic pain of patients could not be evaluated. Sixth, the major limitation of the study is that patients, clinicians, and outcome assessors were not blinded, which may increase the risk of bias and lead to distorted or exaggerated treatment effects. Future studies are recommended to conduct multicenter, randomized controlled prospective trials with expanded sample sizes, and combine psychological assessment tools to comprehensively evaluate the clinical value of hydromorphone PCA in TACE patients.

## Conclusion

To sum up, this study adopted a prospective randomized controlled design to compare the analgesic efficacy and safety of proactive pain management using hydromorphone PCA and traditional as-needed morphine analgesia for perioperative pain management in HCC patients undergoing TACE. Proactive pain management using hydromorphone PCA was associated with lower mean NRS scores at selected time points and with reductions in clinically relevant pain events, cumulative 48-h pain burden, and rescue opioid requirement. However, the absolute between-group differences in mean NRS scores did not clearly exceed the commonly used 1-point clinical-importance threshold and should therefore be interpreted cautiously. No significant between-group differences were observed in short-term quality of life, postoperative length of hospital stay, or willingness to undergo TACE again. Multivariable analysis indicated that complete embolization and as-needed morphine analgesia were significantly associated with breakthrough pain. The hydromorphone PCA strategy was also associated with a higher incidence of dizziness, underscoring the need for individualized dosing and closer safety monitoring. These findings provide clinical evidence for optimizing perioperative pain-management strategies in patients undergoing TACE.

## Data Availability

The original contributions presented in the study are included in the article/Supplementary material, further inquiries can be directed to the corresponding author.
