# Supplementary material for: Efficacy and safety of pain management in perioperative period of TACE in patients with HCC: a single-center, parallel-group, randomized trial
Source: Front Med (Lausanne). 2026 Aug 4;13:1862804. doi: 10.3389/fmed.2026.1862804 (PMC13481502; doi:10.3389/fmed.2026.1862804)
Supplement: Supplementary file 1 [file Table_1.DOCX]

**Supplementary Table S1. Linear mixed-effects model analysis of longitudinal numerical rating scale (NRS) pain scores after transarterial chemoembolization in the hydromorphone patient-controlled analgesia group and the subcutaneous morphine group.**

| **Time point** | **Hydromorphone PCA (n = 51)** | **SCM (n = 54)** | **Time Effect** | | | **Group Effect** | | | **Group × Time Interaction** | | |
| --- | --- | --- | --- | --- | --- | --- | --- | --- | --- | --- | --- |
| **Time point** | **Mean ± SD** | **Mean ± SD** | **F** | **P** | **partial η² (95% CI)** | **F** | **P** | **partial η² (95% CI)** | **F** | **P** | **partial η² (95% CI)** |
| Intraoperative | 2.16 ± 1.43 | 3.09 ± 1.71 | 95.76 | <0.001 | 0.482 (0.412, 0.999) | 10.42 | 0.002 | 0.092 (0.014, 0.999) | 1.38 | 0.240 | 0.013 (0.000, 0.999) |
| 2 h post-TACE | 0.78 ± 1.10 | 1.19 ± 1.59 | 95.76 | <0.001 | 0.482 (0.412, 0.999) | 10.42 | 0.002 | 0.092 (0.014, 0.999) | 1.38 | 0.240 | 0.013 (0.000, 0.999) |
| 12 h post-TACE | 0.51 ± 1.07 | 1.00 ± 1.48 | 95.76 | <0.001 | 0.482 (0.412, 0.999) | 10.42 | 0.002 | 0.092 (0.014, 0.999) | 1.38 | 0.240 | 0.013 (0.000, 0.999) |
| 24 h post-TACE | 0.39 ± 0.67 | 1.04 ± 1.43 | 95.76 | <0.001 | 0.482 (0.412, 0.999) | 10.42 | 0.002 | 0.092 (0.014, 0.999) | 1.38 | 0.240 | 0.013 (0.000, 0.999) |
| 48 h post-TACE | 0.10 ± 0.30 | 0.56 ± 0.96 | 95.76 | <0.001 | 0.482 (0.412, 0.999) | 10.42 | 0.002 | 0.092 (0.014, 0.999) | 1.38 | 0.240 | 0.013 (0.000, 0.999) |
|  |  |  |  |  |  |  |  |  |  |  |  |

Note: *Data were analyzed using a linear mixed-effects model with treatment group, time, and treatment group × time interaction as fixed effects and a subject-specific random intercept. Time was treated as a categorical variable, with the intraoperative assessment used as the reference time category for model parameterization. Model parameters were estimated using restricted maximum likelihood. The model evaluated the overall effects of time, treatment group, and treatment group × time interaction. NRS, numerical rating scale; PCA, patient-controlled analgesia; SCM, subcutaneous morphine; TACE, transarterial chemoembolization.*
